# The blubber adipocyte index: A nondestructive biomarker of adiposity in humpback whales (*Megaptera novaeangliae*)

**DOI:** 10.1002/ece3.2913

**Published:** 2017-06-04

**Authors:** Juliana Castrillon, Wilhelmina Huston, Susan Bengtson Nash

**Affiliations:** ^1^ Southern Ocean Persistent Organic Pollutants Program Environmental Futures Research Institute (EFRI) Griffith University Nathan QLD Australia; ^2^ School of Life Sciences Faculty of Science University of Technology Sydney Ultimo NSW Australia

**Keywords:** adipocyte index, adiposity, Antarctica, body condition, energetic reserves, humpback whales

## Abstract

The ability to accurately evaluate the energetic health of wildlife is of critical importance, particularly under conditions of environmental change. Despite the relevance of this issue, currently there are no reliable, standardized, nonlethal measures to assess the energetic reserves of large, free‐roaming marine mammals such as baleen whales. This study investigated the potential of adipocyte area analysis and further, a standardized adipocyte index (AI), to yield reliable information regarding humpback whale (*Megaptera novaeangliae*) adiposity. Adipocyte area and AI, as ascertained by image analysis, showed a direct correlation with each other but only a weak correlation with the commonly used, but error prone, blubber lipid‐percent measure. The relative power of the three respective measures was further evaluated by comparing humpback whale cohorts at different stages of migration and fasting. Adipocyte area, AI, and blubber lipid‐percent were assessed by binary logistic regression revealing that adipocyte area had the greatest probability to predict the migration cohort with a high level of redundancy attributed to the AI given their strong linear relationship (r = −.784). When only AI and lipid‐percent were assessed, the performance of both predictor variables was significant but the power of AI far exceeded lipid‐percent. The sensitivity of adipocyte metrics and the rapid, nonlethal, and inexpensive nature of the methodology and AI calculation validate the inclusion of the AI in long‐term monitoring of humpback whale population health, and further raises its potential for broader wildlife applications.

## 1 Introduction

The ability to monitor nutritional condition and specifically, the lipid reserves or “adiposity,” of large, free‐roaming cetaceans is essential to estimate energetic health and food availability to a given population (Beck, Smith, & Hammill, 1993). Energetic health has been shown to have a direct influence on diverse areas of mammalian physiology including immune competence, reproduction and offspring survival (Braithwaite, Meeuwig, & Hipsey, 2015; Lockyer, 1987; Loudon, McNeilly, & Milne, 1983; Millar & Hickling, 1990). In capital breeders, such as humpback whales (*Megaptera novaeangliae*), that face the energetic costs of migration and reproduction with energy stores accumulated through intensive summer feeding; the relationship between maternal mass and reproductive success is particularly evident. Indeed, female right whales (*Eubalaena australis*) apparently forego reproduction in years of low food availability (Seyboth et al., 2016). The ability to confidently evaluate maternal adiposity therefore has direct implications for the evaluation of population fecundity and ecosystem productivity (Christiansen, Víkingsson, Rasmussen, & Lusseau, 2014). Whilst measures such as trunk weight, maximum girth, and blubber thickness provide robust indications of energetic reserves in stranded and harvested animals, there are currently no standardized, nonlethal measures that provide reliable information regarding the adiposity of free‐swimming individuals. Blubber lipid content, as measured in shallow biopsy samples, can provide useful information. Lipid analysis is, however, typically performed on sample sizes of <1 g and frequently <0.01 g. Lipid loss from blubber upon dissection is well documented (Krahn et al., 2004, 2007; Ryan, McHugh, O'Connor, & Berrow, 2013), and leads to a high error when handling small sample amounts. Sample handling and approach contributes to the discrepancy observed in the lipid‐percent values obtained from remote biopsy versus necropsy samples described in comparative studies in killer whales (*Orcinus orca*) and beluga whales (*Delphinapterus leucas*) (Krahn et al., 2004), as well as remote versus capture biopsy in polar bears (*Ursus maritimus*) (McKinney et al., 2014). In the latter study, lipid content from remote biopsies were found not to correlate significantly with subjective fatness indices from visual examination. The study concluded that further work is required to establish robust information from lipid content of remote biopsies (McKinney et al., 2014).

In acknowledgement of the importance of this research gap, research efforts have recently been dedicated toward the development of new techniques for the evaluation of energetic health in free‐roaming cetaceans. Promising results have emerged from the application of blubber ultrasounds (Moore et al., 2001) and drone body photography (Christiansen, Dujon, Sprogis, Arnould, & Bejder, 2016; Koski, Abgrall, & Yazvenko, 2010) techniques. There, however, remains a need for routine, comparable and inexpensive measures for broad scale monitoring of both free‐roaming and stranded individuals, facilitating interpopulation comparisons and temporal trend evaluations.

Southern hemisphere humpback whale populations undergo annual cycles of lipid deposition during Antarctic summer feeding, followed by a period of weight loss associated with migration to equatorial breeding ground, fasting, and reproduction. This behavioral life history adaptation is reflected by seasonal changes in blubber thickness, blubber weight, and body girth (Konishi et al., 2008; Lockyer, 1987; Niæss, Haug, & Nilssen, 1998; Vikingsson, 1990; Víkingsson, 1995). In turn, these parameters suggest a change in the physiological state of the whale, with a change in the dynamics of blubber lipid deposition and mobilization (Cropp, Nash, & Hawker, 2014; Miller et al., 2011).

Surplus energy reserves accumulated during summer feeding are stored mainly in the form of lipids in the blubber of the whales (Gómez‐Campos, Borrell, & Aguilar, 2011). Blubber is a modified form of adipose tissue (Waugh, Nichols, Schlabach, Noad, & Bengtson Nash, 2014) unique to marine mammals. It is a type of loose connective tissue comprised of lipid‐filled cells, adipocytes, surrounded by a matrix of collagen fibers, blood vessels, fibroblasts, and immune cells (Ahima & Flier, 2000). The main function of adipocytes is to serve as storage for triglycerides, the main blubber lipid form (Waugh, Schlabach, Noad, & Bengtson Nash, 2012; Waugh et al., 2014). Whilst it has been proposed that the number of adipocytes can be influenced by diet or severe exercise during early life, before or shortly after weaning (Knittle & Hirsch, 1968; Oscai, Spirakis, Wolff, & Beck, 1972; Young, 1976), it is observed that adipocyte number is set early in mammalian development. Thus, under normal conditions, the number of adipocytes remains constant throughout adulthood and fluctuating weight (Faust, Johnson, Stern, & Hirsch, 1978). By extension, this implies that a change in energy stored results in a change in the size or volume of the existing adipocytes, rather than a fluctuation in total adipocyte number. Adipocyte area is, on this basis, expected to act as a semiquantitative measure of mammalian adiposity.

Shallow blubber biopsy collection of skin and outer blubber from free‐roaming as well as stranded individuals is a widely employed sampling technique in cetacean genetic and toxicology research (Bengtson Nash, Waugh, & Schlabach, 2013a). If the outer blubber tissue could further be reliably employed for routine adiposity evaluations, this would represent a significant advancement with minimal effort for implementation, in long‐term population health monitoring programs. This study sought to assess blubber adipocyte metrics for their potential contribute reliable information regarding humpback whale adiposity. The performance of a histological approach in conjunction with image analysis is discussed, and a standardized AI is described and advocated for broad scale implementation.

## 2 Materials and methods

### 2.1 Sample collection

For this study, blubber samples from 203 live biopsied humpback whales were used. Live biopsies were obtained from humpback whales migrating along the east coast of Australia, breeding stock E1, as designated by the International Whaling Commission (IWC, 2001). Animals from this population were sampled off North Stradbroke Island, southeast Queensland (approximately 27°26S, 153°34E) at two different stages of migration, classed at early migration and late migration, respectively. Early migration sampling events took place in June/July when the animals are arriving from their summer feeding grounds and are assumed to have higher relative lipid reserves. Late migration sampling events took place in September/October when the animals were returning to their feeding grounds after several months of fasting, and hence are assumed to have lower relative lipid reserves. The validity of this assumption has previously been demonstrated (Bengtson Nash et al., 2013a). All biopsies were obtained from 6‐m aluminium boat, using hollow‐tipped darts fired from a modified air rifle (Paxarms NZ). The darts were shot at the dorsal region of the whale, ventral, and slightly posterior to the dorsal fin as recommended by Lambertsen, Baker, Weinrich, and Modi (1994). Samples were collected between 2008 and 2016.

### 2.2 Histological preparation

All samples (around 100 mg blubber tissue per sample) were kept frozen until fixed in 10% neutral buffered formalin. Histological preparation followed methods described elsewhere (Parlee, Lentz, Mori, & MacDougald, 2014). Briefly, the blubber tissue was embedded in paraffin and treated with increasing concentrations of alcohol (70, 80, 95, and 100%), in order to gently dehydrate the tissue. It was then cleared with xylene and penetrated with 100% paraffin. The paraffin blocks were sectioned at 5 μm using a rotary microtome and mounted on glass microscope slides. Subsequently, the slides were stained with hematoxylin and eosin (H&E), which stains basophilic structures, showing cytoplasm in pink and nuclei in dark blue. Slides were covered with coverslip using Permount mounting medium.

### 2.3 Image analysis

Slides were viewed with an Olympus BX41 or Olympus BX60 microscope. Digital images were taken with a QImagin MicroPublisher 3.3RTV or Olympus DP72 camera, using QCapture Pro or Olympus DP2‐BSW software. Images were captured covering the entire tissue sample at 10× magnification. For image analysis, the adipocyte‐dominated images were actively selected.

#### 2.3.1 Adipocyte area

**Figure 1:**
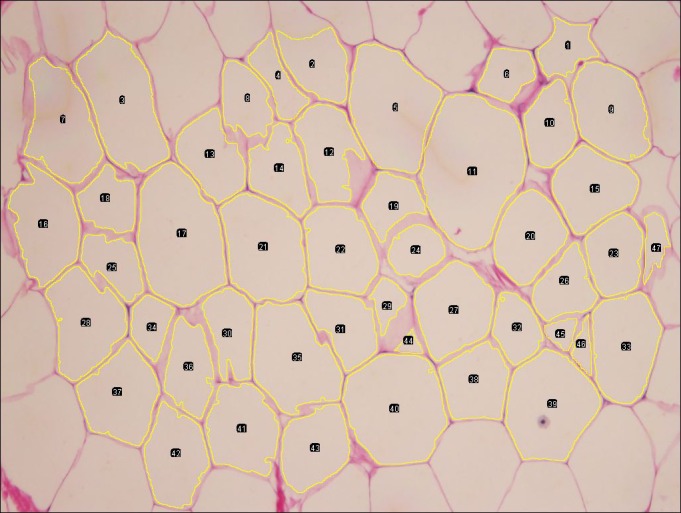
Adipocytes image from blubber biopsy sample. Manual image analysis of adipocyte area using Adiposoft Image Analysis software

Images were analyzed, by manual option, using Open Source Adiposof Image Analysis software (Galarraga et al., 2012), developed as a plug‐in for FIJI (advanced distribution of ImageJ). As it has previously been demonstrated that the same accuracy of data is obtained by analyzing 100, 300, 500, and 1,000 adipocytes (Parlee et al., 2014), a minimum of 100 adipocytes per individual were analyzed. Upon opening of a photographic image, the software automatically, delineates the outline of the adipocytes. Nevertheless, program errors such as missing adipocytes or clustering of two adipocytes were corrected manually. Only complete adipocytes were included for the analysis (Figure 1). The software number each adipocyte and calculate the individual adipocyte cross‐sectional area (from this point on, any reference to area is the cross‐sectional area). The software model assumes a circular shape to the adipocytes and uses the diameter to calculate the area. The software provides default results in pixels, although can be calibrated for other units or conversions made retrospectively. The individual adipocyte area results are used to calculate the average area for the individual sample.

#### 2.3.2 Adipocyte index

To overcome the personnel time investment associated with manual image analysis and in so, facilitate routine, high throughput analysis, we developed and evaluated the adipocyte index (AI), using automated image analysis software. A single representative sample image of ≥167,687.3336 μm^2^ was analyzed using the threshold tool in ImageJ (Abràmoff, Magalhães, & Ram, 2004), a public domain, Java‐based processing program.

In gray scale images, there are 256 (JPG images, 8 bit) or 65,536 (TIF images, 16 bit) intensity graduations which can be assigned to a pixel. A pixel with an intensity of 0 is white and a pixel with a value of 255 or 65,535, for JPG and TIF images respectively, is black.

**Figure 2:**
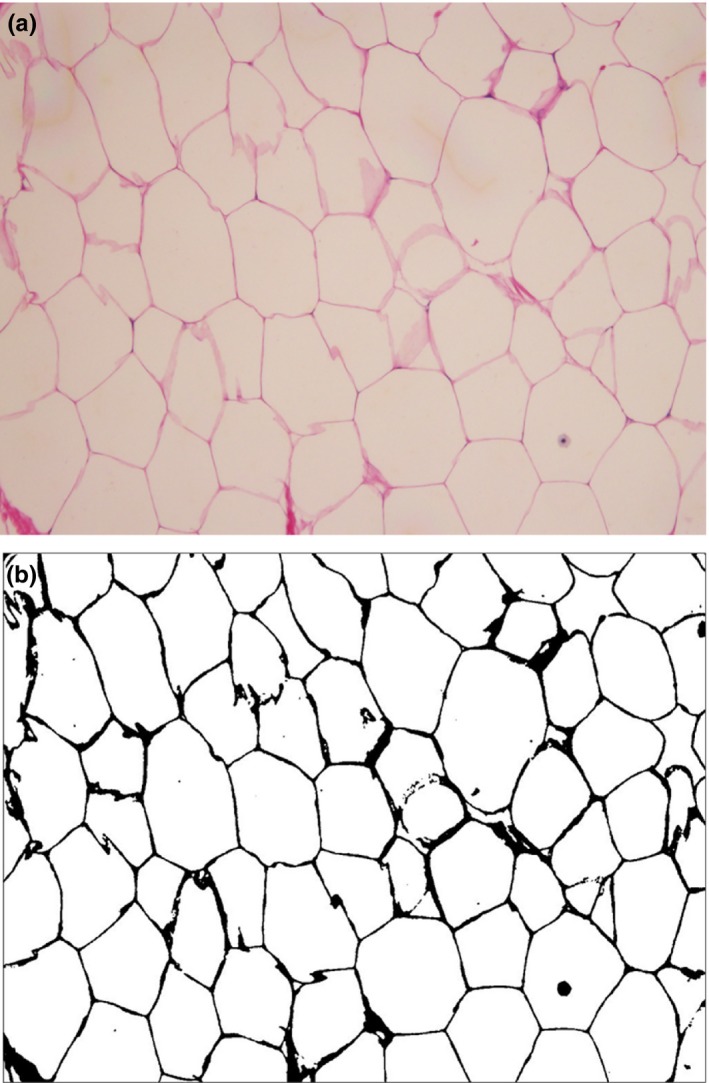
(a) Original adipocyte image stained with H&E. (b) Thresholding methodology applied to the image

Thresholding works by separating pixels which fall within a desired range of intensity values from those that do not (Ferreira & Rasband, 2012). It is the easiest method of image segmentation. From a gray scale, thresholding was used to create binary images (Shapiro & Stockman, 2001), allocating white to lipid‐filled adipocyte area and black to intervacuolar space (Figure 2).

**Figure 3:**
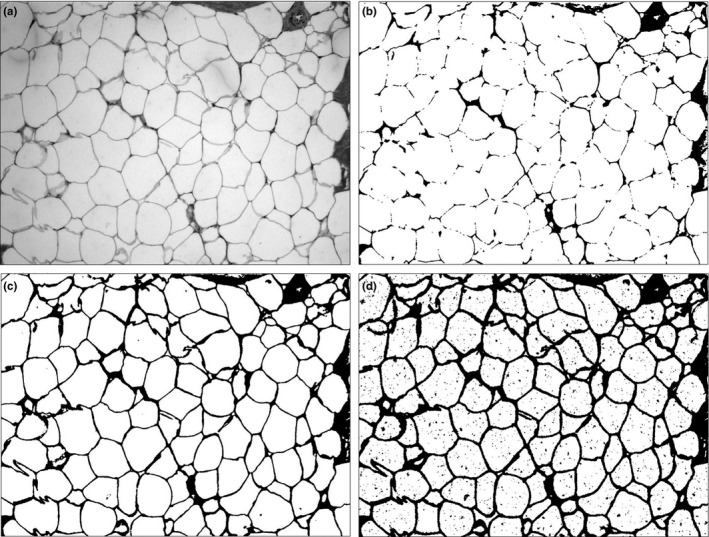
Optimal threshold setup using ImageJ

The threshold set in ImageJ should be set at the discretion of the operator, where the desired results that darkened areas should optimally cover the areas defined as intervacuolar space without covering the adipocyte space (Parlee et al., 2014). Once the optimal threshold has been determined, it must be maintained constant through all the image analysis. For humpback whale blubber in the current study, a threshold value of 65,527 was set for TIF images (Figure 3). The AI is defined as the ratio of intervacuolar area to adipocyte area within the image. Assuming a total image area of 100, a larger adipocyte area (denominator) would yield a smaller AI. Conversely, a higher AI suggests individuals with lower the energy reserves.

In order to evaluate the agreement between manual adipocyte area image analysis and the AI based on automated analysis, a total of 84 samples were analyzed by both methodologies (Table 1).

**Table 1:** The number of humpback whale biopsy samples analyzed per fasting cohort, displayed with the adipocyte area geometric mean and range

Blubber metric	Number of samples		Geometric mean	Range
	Early migration	Late migration		
Ad. area	42	41	660.89 μm^2^	283.79–1,172 μm^2^
AI	98	105	1.42	1–1.83
Lipid %	38	101	39.335	2.81–77.656

#### 2.3.3 Lipid analyses

All samples were analyzed for total lipids. Preweighed blubber tissue samples (~ 0.005 to 0.05 g) were extracted overnight using a modified Bligh and Dyer DCM/methanol/water (1:2:0.8 v/v/v) extraction. Addition of DCM/water the following day (final ratio 1:1:0.9 v/v/v) allowed the samples to separate into aqueous and DCM phases. The lipids were then extracted from the lower phase and trans‐methylated and reduced by rotary evaporation to obtain the total lipid extract. Full methods for lipid analysis are presented in Waugh, Nichols, Noad, and Bengtson Nash (2012). (For adipocyte area, AI and lipid‐percent results see supplementary information)

#### 2.3.4 Statistical analysis

Adipocyte area and lipid‐percent data were found to be normally distributed, using Shapiro–Wilk test (*p *= .196 and *p* = .203, respectably). Homogeneity of variances was verified by the Levene test (*p* = .783, *p* = .082, and *p* = .499, for adipocyte area, AI, and lipid‐percent, respectably). AI data were not normally distributed (*p* = .001), and Log10 transformations were performed prior to statistical analysis (AI Log10, *p* = .758). Not outliers were detected using 2.2 interquartile range (IQR). Collinearity between the variables was investigated using variance inflation factor (VIF), all the variables had a VIF value below three, suggesting no collinearity within the variables. For statistical comparison, an independent sample student *t* test was used to compare average lipid‐percent, adipocyte area, and AI between early and late migrations. Pearson correlation coefficients were applied to correlate adipocyte area and AI, adipocyte area and lipid‐percent, and AI and lipid‐percent. Binary logistic regression models were used to assess the ability of the independent adiposity measure variables (adipocyte area, blubber lipid‐percent, and AI) to predict the response variable (migration cohort). The original AI data set (not normally distributed) was used for this analysis, as normality is not a prerequisite for binomial logistic regression application. In the logistic regression models, a backward likelihood ratio (LR) was used as a variable selection method. This method starts with a model in which all the variables are included and removal testing of the variables is based on the probability of the likelihood‐ratio statistic based on the maximum partial likelihood estimates. The predictive power of the models was evaluated with Nagelkerke R Square and the goodness of fit with Hosmer and Lemeshow. Statistics were conducted using IBM SPSS (v.22 IBM SPSS Inc., Chicago, IL, USA).

## 3 Results

### 3.1 Adipocyte metrics and lipid percent

Histological analysis of outer blubber found an average adipocyte area of 792.10 μm^2^ (range: 283.79–1,172.00), for early migration animals and of 597.65 μm^2^ (range: 336.27–891.91) for late migration animals (total average adipocyte area was 690.99 μm^2^ [range: 782.10–597.65]).These areas correspond to adipocyte volumes of 0.019527 and 0.007516 nl, in early and late migration, respectively (Table 1), assuming a spherical adipocyte shape.

As expected, given the common source images, the AI presented a strong negative correlation with the adipocyte area measurements on the same individuals (r = −.784, *p *= <.001).

**Figure 4:**
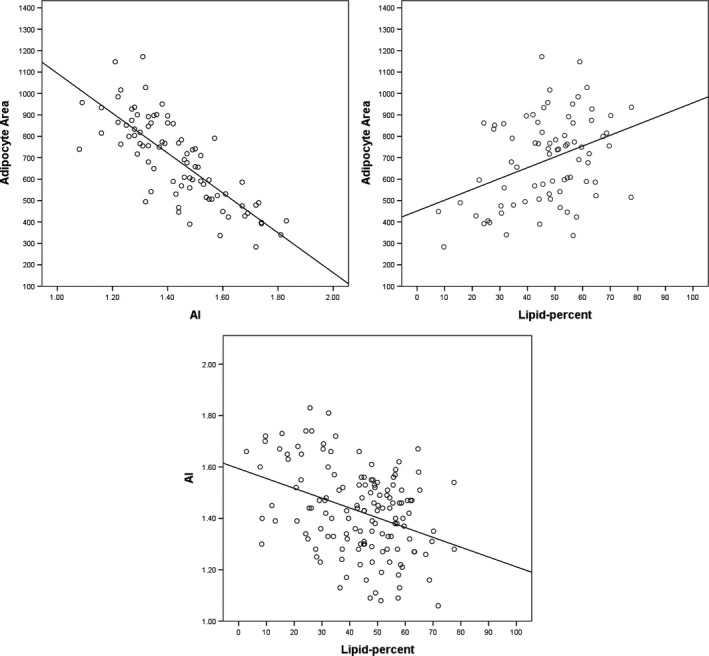
Correlation between the different adipocyte metrics

This finding supports the ability of the AI to adequately simulate manual adipocyte area measurements. When adipocyte area and AI were compared with outer blubber lipid‐percent (wet weight), the commonly measured parameter used to indicate relative energy reserves in both stranded and free‐ranging individuals, blubber lipid was found to have moderately positive correlation with adipocyte area (r = .388, *p *= <.001), and similar correlation but negative with AI (r = −.468, *p *= <.001) (Figure 4).

When all free‐ranging individuals were grouped according to migration cohort, differentiation by lipid‐percent was not possible (*p *=* *.884, with 95% confident). By contrast, both adipocyte area and AI were sufficiently sensitive to detect this difference in means (*p *= <.001 and *p *=* *.002).

In order to further evaluate the power of the respective adiposity measures, their ability to predict the migratory cohort of an individual was evaluated using binary logistic regressions models with backwards LR as a variable selection method (Table 2). In the four (A–D) models applied, the response variables were the migration cohorts and the predictors were the adiposity measures, namely, adipocyte area, AI, and lipid‐percent, respectively. When a logistic regression was performed including all three predictors (model A) a surprising result was observed, adipocyte area contribution to the model was not significant (Table 3). In this model 62% of the data was lost, as just the samples with the three measurements were suitable for the analysis. Additionally, in logistic regression, collinearity among the predictors can lead to biased estimates. On this basis, we suspected that the model is affected to some degree by the high correlation between adipocyte area and AI, and by smaller sample size, which leads it to omit adipocyte area as model predictor. When the same model (B) was run with just AI and lipid‐percent as predictors, the variable selection method estimated that lipid‐percent was not a significant predictor in the model, recognizing that the best model to predict the migration cohort was the model with just AI as a predictor. In model C, the predictors included in the analysis were adipocyte area and lipid‐percent. The result shows that despite the reduction in adipocyte area sample size, both of the predictors were deemed important in the model. Finally, model D, used the predictors adipocyte area and AI. Once again adipocyte area did not contribute significantly to the model, confirming that the correlation between these two predictors confounded results.

**Table 2:** A description of the logistic regression model parameters used, showing the model, the number of samples used for each model, the variable selection steps, the predictors and response in each model, the standard error, *t*, the significance, the odd ratio, and the coefficient confidence intervals

Logistic regression models										
Model	N	Steps	Predictors	Response	B coefficient	S.E.	Sig.	Odd ratio	95% C.I.	
									Lower	Upper
A	77	Step 1	Ad. area	Early migration = 0 Late migration = 1	0.000	0.000	0.221	1.000	0.999	1.000
			AI		0.151	0.051	0.003	1.163	1.053	1.285
			Lipid %		0.075	0.026	0.004	1.078	1.024	1.135
		Step 2	AI		0.190	0.044	0.000	1.210	1.110	1.318
			Lipid %		0.074	0.026	0.005	1.077	1.023	1.133
B	139	Step 1	AI	Early migration = 0 Late migration = 1	0.101	0.026	0.000	1.106	1.052	1.163
			Lipid %		0.002	0.014	0.863	1.002	0.975	1.031
		Step 2	AI		0.100	0.025	0.000	1.105	1.053	1.159
C	77	Step 1	Lipid %	Early migration = 0 Late migration = 1	0.043	0.021	0.042	1.044	1.002	1.088
			Ad. area		0.000	0.000	0.000	0.999	0.999	1.000
D	83	Step 1	Ad. area	Early migration = 0 Late migration = 1	0.000	0.000	0.237	1.000	0.999	1.000
			AI		0.090	0.038	0.017	1.094	1.016	1.177
		Step 2	AI		0.119	0.030	0.000	1.127	1.063	1.194
E	77	Step 1	Ad. area	Early migration = 0 Late migration = 1	0.000	0.000	0.000	0.999	0.999	1.000
F	77	Step 1	AI		0.129	0.032	0.000	1.138	1.069	1.212
G	77	Step 1	Lipid %		0.001	0.015	0.971	1.001	0.971	1.031

**Table 3:** The predictive power of the binary regression models as evaluated through Nagelkerke R Square and the goodness of fit of each model, using Hosmer and Lemeshow

Predictive power			Goodness of fit		
			Hosmer & Lemeshow test		
Model	Predictors	Nagelkerke R square	Chi‐square	*df*	Sig.
A	AI Lipid %	0.503	8.545	8	0.382
B	AI	0.221	8.517	8	0.385
C	Lipid % Ad. area	0.378	4.206	8	0.838
D	AI	0.346	3.383	8	0.908
E	Ad. area	0.32	5.36	8	0.719
F	AI	0.391	8.362	8	0.399
G	Lipid %	0	7.815	8	0.452

As all the predictors were correlated in some way, univariate analysis was performed. Three extra models were run, with each measurement as a single predictor (models E, F, and G). In models E and F, both adipocyte area and AI were significant in isolation. By contrast, model G, which used lipid‐percent as a single predictor, was not significant, corroborating finding that this measurement needs to be complemented in the model by other predictors. According to the predictive power, the better models at predicting the migration cohorts were model A (AI and lipid‐percent) and model F (AI). All models fit the data according to the goodness of fit test (Table 3).

Where data from both states of fasting were available, it was possible to quantify an adipocyte size reduction of 30% between early and late migration. If blubber lipid volume reduction is a reliable approximation of the corresponding reduction in blubber mass, this equates a 6.7% reduction in whale body mass between the two migration cohorts, as calculated using the empirical tissue weights applied in Bengtson Nash, Waugh, and Schlabach (2013b).

## 4 Discussion

The adipocyte area analysis and AI data presented in this study, showed only a weak correlation with one of the few, nondestructive, measures commonly used to indicate adiposity in both free‐roaming and stranded animals. In the absence of controlled experimentation or gross morphological assessment, logistically prohibited in free‐swimming animals, it remained unclear which of the three proposed adiposity measures provided the most accurate information regarding individual adiposity. To further evaluate this, the power of each measure in predicting the migration cohort, and therefore fasting state, of individuals, was investigated. Whilst adipocyte area was found to be the most sensitive measure in distinguishing between the means of the cohorts, its inclusion in a logistic regression model alongside the two other adiposity measures did not verify its power as a predictor variable. Upon further examination, this could be attributed to its strong collinearity with AI, or the redundancy between the two measures. In isolation, adipocyte area carried significant power. Results of logistic regression found that AI in conjunction with lipid‐percent provide the strongest predicting power, follow by AI in isolation. Lipid‐percent was found to neither carry significant predictive power in the model, nor be sufficiently sensitive to separate cohorts by means.

Interpretation of all outer blubber adiposity measures, however, requires consideration of the inherent limitations of this sampled tissue. Blubber in cetaceans differs in biochemical composition and in function, along its depth (Lockyer, McConnell, & Waters, 1984; Strandberg et al., 2008). In addition to energy storage, the outer blubber plays a number of important ancillary roles which may place a threshold upon maximum lipid loss from this layer. External blubber is associated with thermoregulation, buoyancy, water balance, and locomotion, among other important physiological functions (Koopman, Pabst, McLellan, Dillaman, & Read, 2002; Ryg, Smith, & Øritsland, 1988). Loss of too much lipid from this region would therefore compromise these ancillary functions and hereby jeopardise individual survival. It is therefore not expected that a linear relationship between lipid loss from the outer blubber layer and whole‐of‐body adiposity exists, as indicated by the findings of Bengtson Nash et al. (2013b). This places an inherent limitation on the power of both blubber lipid‐percent and adipocyte metrics. Nonetheless, in this study, the observed fluctuations in adipocyte measures were sufficient to correctly categorize animals according to their migration/fasting cohort. This finding suggests significant potential for adipocyte metrics to be applied for the purpose of investigating temporal trends and interpopulation differences in long‐term monitoring programs.

## 5 Conclusion

Despite the importance of determining the energetic health of free‐roaming cetaceans, reliable, nondestructive techniques are lacking. A wider set of biomarkers is necessary to accurately ascertain energetic health. The AI, offers a new, practical and low‐cost, measure of relative energy reserves within a population to support ongoing population health research and monitoring activities.

## AutHor contributions

JC WH SBN conceived and designed the experiments; JC performed the experiments; JC WH SBN analyzed the data; JC WH SBN wrote the manuscript.

## Conflict of interest

None declared.
