# Supplementary material for: The blubber adipocyte index: A nondestructive biomarker of adiposity in humpback whales (Megaptera novaeangliae)
Source: Ecol Evol. 2017 Jun 4;7(14):5131–9. doi: 10.1002/ece3.2913 (PMC5528216; doi:10.1002/ece3.2913)
Supplement: Supplementary file 1 [file ECE3-7-5131-s001.docx]

**Supplementary data.**

**Table S1:** Individual label, the year in where the sample were taken, the migration cohort, number of adipocytes analysed for each individual, adipocyte area in microns, adipocyte index and lipid percentage.

| **Label** | **Year** | **Migration** | **Adipocytes** | **Ad. Area in µm** | **AI** | **Lipid %** |
| --- | --- | --- | --- | --- | --- | --- |
| 10N08 | 2008 | Early Migration | 119 | 13826.818 | 15.7 | 58.947 |
| 11N08 | 2008 | Early Migration | 112 | 10850.554 | 19.05 | - |
| 17N08 | 2008 | Early Migration | 127 | 9219.674 | 20.62 | 43.964 |
| 20N08 | 2008 | Early Migration | 106 | 14116.178 | 22.2 | 45.197 |
| 22N08 | 2008 | Early Migration | 142 | 11861.525 | 16.04 | 58.333 |
| 24N08 | 2008 | Early Migration | 135 | 12241.443 | 16.82 | 48.092 |
| 26N08 | 2008 | Early Migration | 105 | 10040.466 | 18.01 | 27.763 |
| 4N09 | 2009 | Early Migration | 150 | 5405.933 | 38.23 | 7.759 |
| 11N09 | 2009 | Early Migration | 219 | 3418.111 | 50.95 | 9.697 |
| 1N14 | 2014 | Early Migration | 155 | 5315.204 | 43.17 | 30.587 |
| 3N14 | 2014 | Early Migration | 109 | 9258.618 | 20.65 | 42.795 |
| 6N14 | 2014 | Early Migration | 117 | 11246.911 | 14.06 | 45.936 |
| 7N14 | 2014 | Early Migration | 154 | 6387.463 | 26.4 | - |
| 8N14 | 2014 | Early Migration | 112 | 8551.376 | 33.24 | - |
| 9N14 | 2014 | Early Migration | 108 | 10259.521 | 17.54 | 28.095 |
| 10N14 | 2014 | Early Migration | 128 | 8192.769 | 22.6 | 34.168 |
| 13N14 | 2014 | Early Migration | 124 | 7926.454 | 31.65 | - |
| 15N14 | 2014 | Early Migration | 111 | 9527.198 | 37.06 | 34.464 |
| 16N14 | 2014 | Early Migration | 111 | 9680.267 | 18.78 | 53.491 |
| 17N14 | 2014 | Early Migration | 113 | 8656.100 | 27.99 | 62.406 |
| 18N14 | 2014 | Early Migration | 103 | 11169.263 | 13.99 | 63.356 |
| 19N14 | 2014 | Early Migration | 125 | 9815.459 | 14.21 | 68.702 |
| 20N14 | 2014 | Early Migration | 125 | 9235.178 | 20.26 | 48.092 |
| 21N14 | 2014 | Early Migration | 125 | 10787.123 | 19.71 | 39.594 |
| 22N14 | 2014 | Early Migration | 120 | 8877.279 | 24.83 | 50.809 |
| 23N14 | 2014 | Early Migration | 109 | 11532.721 | 14.02 | 47.363 |
| 24N14 | 2014 | Early Migration | 106 | 9315.530 | 23.68 | 57.003 |
| 26N14 | 2014 | Early Migration | 137 | 9105.802 | 18.85 | 53.847 |
| 27N14 | 2014 | Early Migration | 112 | 10385.197 | 21.52 | 24.167 |
| 28N14 | 2014 | Early Migration | 129 | 7899.791 | 27.64 | 36.196 |
| 29N14 | 2014 | Early Migration | 103 | 10854.520 | 22.37 | 42.012 |
| 30N14 | 2014 | Early Migration | 115 | 9438.081 | 28.58 | 50.212 |
| 31N14 | 2014 | Early Migration | 113 | 9860.583 | 20.15 | 45.308 |
| 32N14 | 2014 | Early Migration | 175 | 6520.437 | 20.89 | 51.741 |
| 33N14 | 2014 | Early Migration | 163 | 6735.985 | 30.37 | 31.573 |
| 34N14 | 2014 | Early Migration | 141 | 11450.071 | 23.89 | 56.378 |
| 35N14 | 2014 | Early Migration | 110 | 10801.621 | 23.25 | 70.21 |
| 36N14 | 2014 | Early Migration | 126 | 12378.112 | 20.77 | 61.588 |
| 37N14 | 2014 | Early Migration | 135 | 8907.055 | 11.83 | 51.236 |
| 38N14 | 2014 | Early Migration | 103 | 11267.573 | 18.84 | 77.656 |
| 39N14 | 2014 | Early Migration | 144 | 6203.506 | 21.95 | 77.556 |
| 40N14 | 2014 | Early Migration | 142 | 7118.191 | 33.51 | 49.1 |
| 12S08 | 2008 | Late Migration | 100 | 10343.507 | 24.89 | 31.38 |
| 15S08 | 2008 | Late Migration | 131 | 9192.588 | 15.84 | 54.386 |
| 23S08 | 2008 | Late Migration | 161 | 5160.120 | 45.99 | 21.344 |
| 1S09 | 2009 | Late Migration | 147 | 7820.891 | 21.61 | - |
| 3S09 | 2009 | Late Migration | 178 | 6106.207 | 33.93 | 48.337 |
| 4S09 | 2009 | Late Migration | 131 | 9095.086 | 19.57 | 69.634 |
| 6S09 | 2009 | Late Migration | 102 | 10200.930 | 23.71 | - |
| 11S09 | 2009 | Late Migration | 102 | 10535.341 | 17.92 | 63.203 |
| 15S09 | 2009 | Late Migration | 114 | 9025.898 | 22.89 | 59.593 |
| 7S11 | 2011 | Late Migration | 118 | 5768.960 | 51 | 34.945 |
| 8S11 | 2011 | Late Migration | 149 | 4883.335 | 55.24 | 25.693 |
| 11S11 | 2011 | Late Migration | 126 | 5897.233 | 53.43 | 15.633 |
| 23S11 | 2011 | Late Migration | 137 | 4097.103 | 66.01 | 32.381 |
| 24S11 | 2011 | Late Migration | 111 | 5722.345 | 45.26 | 30.43 |
| 2S13 | 2013 | Late Migration | 147 | 7092.895 | 31.34 | 61.37 |
| 3S13 | 2013 | Late Migration | 156 | 6942.774 | 32.75 | 45.59 |
| 5S13 | 2013 | Late Migration | 146 | 7329.001 | 28.4 | 55.53 |
| 6S13 | 2013 | Late Migration | 179 | 6101.582 | 36.07 | 44.2 |
| 7S13 | 2013 | Late Migration | 158 | 4789.038 | 55.12 | 26.32 |
| 8S13 | 2013 | Late Migration | 102 | 10389.919 | 41.48 | 56.52 |
| 9S13 | 2013 | Late Migration | 123 | 10742.649 | 41.44 | 54.92 |
| 10S13 | 2013 | Late Migration | 209 | 4698.791 | 38.66 | 44.44 |
| 11S13 | 2013 | Late Migration | 100 | 7057.756 | 47.61 | 64.59 |
| 12S13 | 2013 | Late Migration | 143 | 8151.977 | 29.01 | 61.93 |
| 13S13 | 2013 | Late Migration | 197 | 5629.713 | 30.25 | 51.85 |
| 14S13 | 2013 | Late Migration | 241 | 4050.186 | 49.46 | 56.55 |
| 15S13 | 2013 | Late Migration | 177 | 4721.627 | 55.77 | 24.22 |
| 16S13 | 2013 | Late Migration | 140 | 6393.586 | 40.87 | 47.89 |
| 17S13 | 2013 | Late Migration | 145 | 6847.941 | 27.01 | 42.62 |
| 20S13 | 2013 | Late Migration | 152 | 7190.742 | 30.21 | 53.38 |
| 21S13 | 2013 | Late Migration | 171 | 5958.726 | 37.82 | 39.13 |
| 22S13 | 2013 | Late Migration | 189 | 5098.332 | 40.81 | 57.78 |
| 26S13 | 2013 | Late Migration | 111 | 9632.408 | 17.47 | 67.41 |
| 28S13 | 2013 | Late Migration | 208 | 5370.517 | 26.74 | 54.42 |
| 29S13 | 2013 | Late Migration | 117 | 8323.319 | 28.08 | 58.64 |
| 31S13 | 2013 | Late Migration | 132 | 7180.886 | 35.39 | 22.5 |
| 33S13 | 2013 | Late Migration | 121 | 10424.705 | 26.85 | 43.76 |
| 34S13 | 2013 | Late Migration | 138 | 8639.923 | 27.77 | 47.95 |
| 35S13 | 2013 | Late Migration | 133 | 7294.384 | 29.78 | 54.41 |
| 36S13 | 2013 | Late Migration | 110 | 8933.110 | 31.56 | 47.54 |
| 37S13 | 2013 | Late Migration | 149 | 6298.322 | 39.95 | 64.83 |
| 1S15 | 2015 | Late Migration | - | - | 35.61 | 55.6567 |
| 2S15 | 2015 | Late Migration | - | - | 41.02 | 47.8053 |
| 3S15 | 2015 | Late Migration | - | - | 15.31 | 36.5357 |
| 4S15 | 2015 | Late Migration | - | - | 19.71 | 37.1851 |
| 5S15 | 2015 | Late Migration | - | - | 14.36 | 57.4292 |
| 6S15 | 2015 | Late Migration | - | - | 16.75 | 57.5686 |
| 7S15 | 2015 | Late Migration | - | - | 24.77 | 48.0213 |
| 8S15 | 2015 | Late Migration | - | - | 44.46 | 32.218 |
| 9S15 | 2015 | Late Migration | - | - | 23.03 | 45.088 |
| 10S15 | 2015 | Late Migration | - | - | 53.82 | 9.55096 |
| 11S15 | 2015 | Late Migration | - | - | 35.15 | 20.7143 |
| 12S15 | 2015 | Late Migration | - | - | 32.85 | 29.1001 |
| 13S15 | 2015 | Late Migration | - | - | 36.4 | 31.0996 |
| 14S15 | 2015 | Late Migration | - | - | 25.57 | 13.2368 |
| 15S15 | 2015 | Late Migration | - | - | 31.51 | 49.9288 |
| 16S15 | 2015 | Late Migration | - | - | 21.65 | 8.32145 |
| 17S15 | 2015 | Late Migration | - | - | 40.13 | 56.3575 |
| 18S15 | 2015 | Late Migration | - | - | 14.18 | 49.3052 |
| 19S15 | 2015 | Late Migration | - | - | 36.19 | 37.5542 |
| 20S15 | 2015 | Late Migration | - | - | 38.88 | 45.2035 |
| 21S15 | 2015 | Late Migration | - | - | 45.6 | 17.8225 |
| 22S15 | 2015 | Late Migration | - | - | 19.59 | 37.3073 |
| 23S15 | 2015 | Late Migration | - | - | 50.41 | 22.6065 |
| 24S15 | 2015 | Late Migration | - | - | 13.62 | 71.8589 |
| 25S15 | 2015 | Late Migration | - | - | 23.41 | 43.1831 |
| 26S15 | 2015 | Late Migration | - | - | 32.16 | 60.8119 |
| 27S15 | 2015 | Late Migration | - | - | 35.72 | 65.2551 |
| 28S15 | 2015 | Late Migration | - | - | 48.08 | 33.2268 |
| 29S15 | 2015 | Late Migration | - | - | 35.57 | 48.9396 |
| 30S15 | 2015 | Late Migration | - | - | 29.48 | 25.3968 |
| 31S15 | 2015 | Late Migration | - | - | 19.28 | 51.4205 |
| 32S15 | 2015 | Late Migration | - | - | 55.11 | 43.413 |
| 33S15 | 2015 | Late Migration | - | - | 36.78 | 38.8181 |
| 34S15 | 2015 | Late Migration | - | - | 22.85 | 32.1695 |
| 35S15 | 2015 | Late Migration | - | - | 26.51 | 29.5734 |
| 37S15 | 2015 | Late Migration | - | - | 27.27 | 21.0047 |
| 38S15 | 2015 | Late Migration | - | - | 20.47 | 29.4155 |
| 39S15 | 2015 | Late Migration | - | - | 23.76 | 24.9582 |
| 40S15 | 2015 | Late Migration | - | - | 28.3 | 11.9829 |
| 41S15 | 2015 | Late Migration | - | - | 31.81 | 57.9344 |
| 42S15 | 2015 | Late Migration | - | - | 31.06 | 38.9091 |
| 43S15 | 2015 | Late Migration | - | - | 27.93 | 8.46774 |
| 44S15 | 2015 | Late Migration | - | - | 41.63 | 45.1663 |
| 45S15 | 2015 | Late Migration | - | - | 39.86 | 55.7819 |
| 46S15 | 2015 | Late Migration | - | - | 13.11 | 57.9613 |
| 47S15 | 2015 | Late Migration | - | - | 18 | 51.8771 |
| 48S15 | 2015 | Late Migration | - | - | 23.83 | 33.529 |
| 50S15 | 2015 | Late Migration | - | - | 30.83 | 53.6224 |
| 52S15 | 2015 | Late Migration | - | - | 12.92 | 38.8552 |
| 53S15 | 2015 | Late Migration | - | - | 30.39 | 26.1596 |
| 54S15 | 2015 | Late Migration | - | - | 30.62 | 48.7034 |
| 55S15 | 2015 | Late Migration | - | - | 60.9 | - |
| 56S15 | 2015 | Late Migration | - | - | 50.64 | 17.5125 |
| 57S15 | 2015 | Late Migration | - | - | 23.25 | 59.1981 |
| 58S15 | 2015 | Late Migration | - | - | 36 | 43.4016 |
| 59S15 | 2015 | Late Migration | - | - | 35.62 | 49.9473 |
| 60S15 | 2015 | Late Migration | - | - | 19.58 | 57.2009 |
| 61S15 | 2015 | Late Migration | - | - | 29.19 | 45.2502 |
| 62S15 | 2015 | Late Migration | - | - | 21.26 | 49.1569 |
| 63S15 | 2015 | Late Migration | - | - | 33.28 | 58.6994 |
| 64S15 | 2015 | Late Migration | - | - | 61.22 | - |
| 65S15 | 2015 | Late Migration | - | - | 47.41 | 14.7739 |
| 66S15 | 2015 | Late Migration | - | - | 23.15 | 43.7931 |
| 67S15 | 2015 | Late Migration | - | - | 46.5 | 2.81501 |
| 1N16 | 2016 | Early Migration | - | - | 32.22 | - |
| 4N16 | 2016 | Early Migration | - | - | 38.11 | - |
| 5N16 | 2016 | Early Migration | - | - | 25.23 | - |
| 6N16 | 2016 | Early Migration | - | - | 30.72 | - |
| 7N16 | 2016 | Early Migration | - | - | 45.5 | - |
| 8N16 | 2016 | Early Migration | - | - | 32.38 | - |
| 9N16 | 2016 | Early Migration | - | - | 31.69 | - |
| 10N16 | 2016 | Early Migration | - | - | 32.7 | - |
| 11N16 | 2016 | Early Migration | - | - | 40.81 | - |
| 12N16 | 2016 | Early Migration | - | - | 34.98 | - |
| 13N16 | 2016 | Early Migration | - | - | 37.18 | - |
| 14N16 | 2016 | Early Migration | - | - | 28.11 | - |
| 15N16 | 2016 | Early Migration | - | - | 24.67 | - |
| 16N16 | 2016 | Early Migration | - | - | 33.63 | - |
| 17N16 | 2016 | Early Migration | - | - | 17.53 | - |
| 18N16 | 2016 | Early Migration | - | - | 36.72 | - |
| 19N16 | 2016 | Early Migration | - | - | 19.15 | - |
| 21N16 | 2016 | Early Migration | - | - | 37.09 | - |
| 22N16 | 2016 | Early Migration | - | - | 30.38 | - |
| 24N16 | 2016 | Early Migration | - | - | 24.45 | - |
| 25N16 | 2016 | Early Migration | - | - | 19.84 | - |
| 26N16 | 2016 | Early Migration | - | - | 38.58 | - |
| 27N16 | 2016 | Early Migration | - | - | 30.48 | - |
| 28N16 | 2016 | Early Migration | - | - | 37.26 | - |
| 30N16 | 2016 | Early Migration | - | - | 22.33 | - |
| 31N16 | 2016 | Early Migration | - | - | 29.74 | - |
| 32N16 | 2016 | Early Migration | - | - | 19.28 | - |
| 33N16 | 2016 | Early Migration | - | - | 32.29 | - |
| 34N16 | 2016 | Early Migration | - | - | 45.78 | - |
| 35N16 | 2016 | Early Migration | - | - | 10.46 | - |
| 36N16 | 2016 | Early Migration | - | - | 21.77 | - |
| 37N16 | 2016 | Early Migration | - | - | 23.99 | - |
| 38N16 | 2016 | Early Migration | - | - | 30.63 | - |
| 39N16 | 2016 | Early Migration | - | - | 35.63 | - |
| 40N16 | 2016 | Early Migration | - | - | 34.72 | - |
| 41N16 | 2016 | Early Migration | - | - | 19.08 | - |
| 42N16 | 2016 | Early Migration | - | - | 41.79 | - |
| 43N16 | 2016 | Early Migration | - | - | 30.14 | - |
| 44N16 | 2016 | Early Migration | - | - | 32.72 | - |
| 45N16 | 2016 | Early Migration | - | - | 29.82 | - |
| 46N16 | 2016 | Early Migration | - | - | 46.7 | - |
| 47N16 | 2016 | Early Migration | - | - | 44.9 | - |
| 48N16 | 2016 | Early Migration | - | - | 51.47 | - |
| 49N16 | 2016 | Early Migration | - | - | 16.06 | - |
| 50N16 | 2016 | Early Migration | - | - | 13.71 | - |
| 52N16 | 2016 | Early Migration | - | - | 31.58 | - |
| 53N16 | 2016 | Early Migration | - | - | 13.2 | - |
| 54N16 | 2016 | Early Migration | - | - | 38.13 | - |
| 55N16 | 2016 | Early Migration | - | - | 23.4 | - |
| 56N16 | 2016 | Early Migration | - | - | 34.14 | - |
| 57N16 | 2016 | Early Migration | - | - | 42.4 | - |
| 58N16 | 2016 | Early Migration | - | - | 23.19 | - |
| 59N16 | 2016 | Early Migration | - | - | 16.36 | - |
| 60N16 | 2016 | Early Migration | - | - | 27.09 | - |
| 61N16 | 2016 | Early Migration | - | - | 14.45 | - |
| 62N16 | 2016 | Early Migration | - | - | 51.69 | - |
